# Supplementary material for: Identification of acquired Notch3 dependency in metastatic Head and Neck Cancer
Source: Commun Biol. 2023 May 18;6:538. doi: 10.1038/s42003-023-04828-9 (PMC10195806; doi:10.1038/s42003-023-04828-9)
Supplement: Supplementary file 2 — Supplementary Figures [file 42003_2023_4828_MOESM2_ESM.pdf]

a.

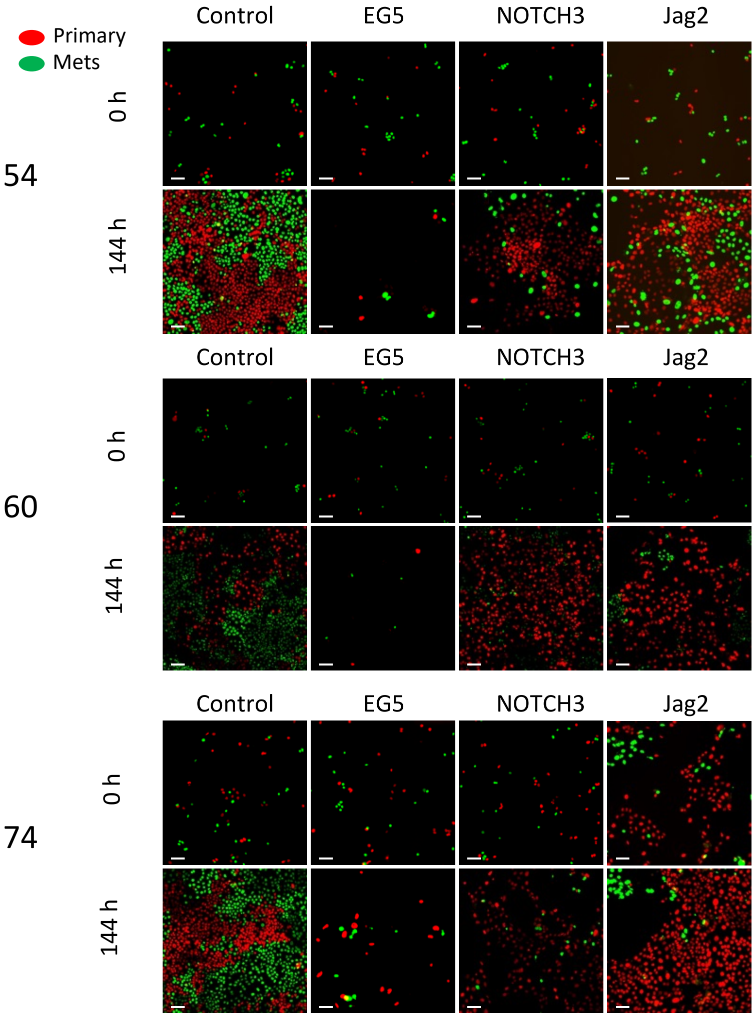

b.

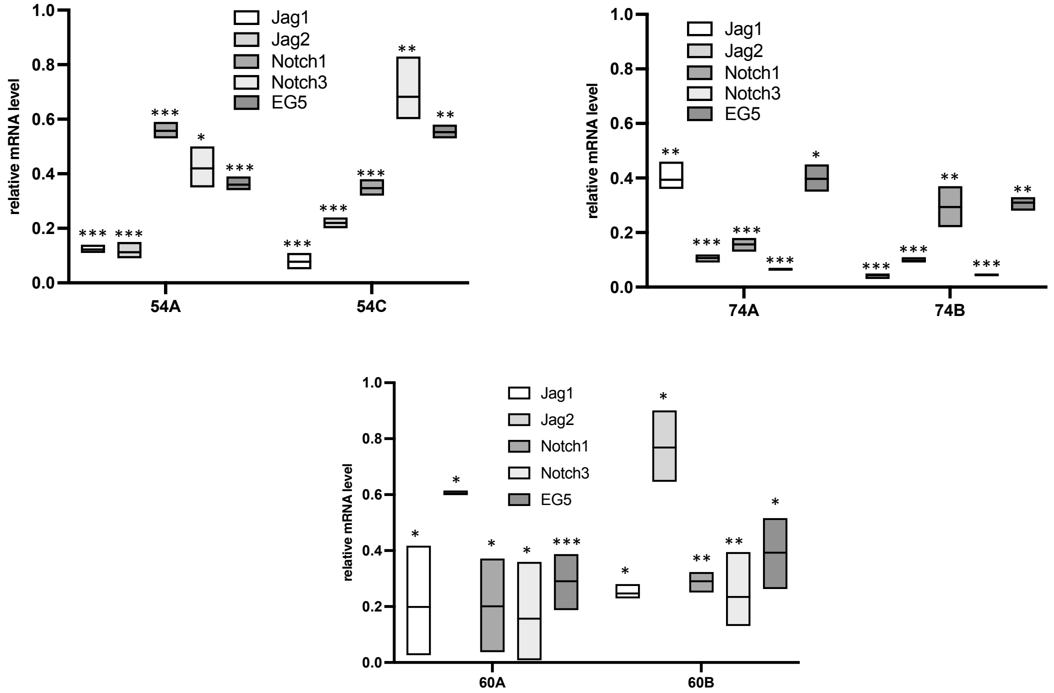

Figure S1. High throughput fluorescent imaging was utilized to evaluate the effects of knocking down of Notch3 on the growth of HNSCC cells derived from primary tumors and metastases.

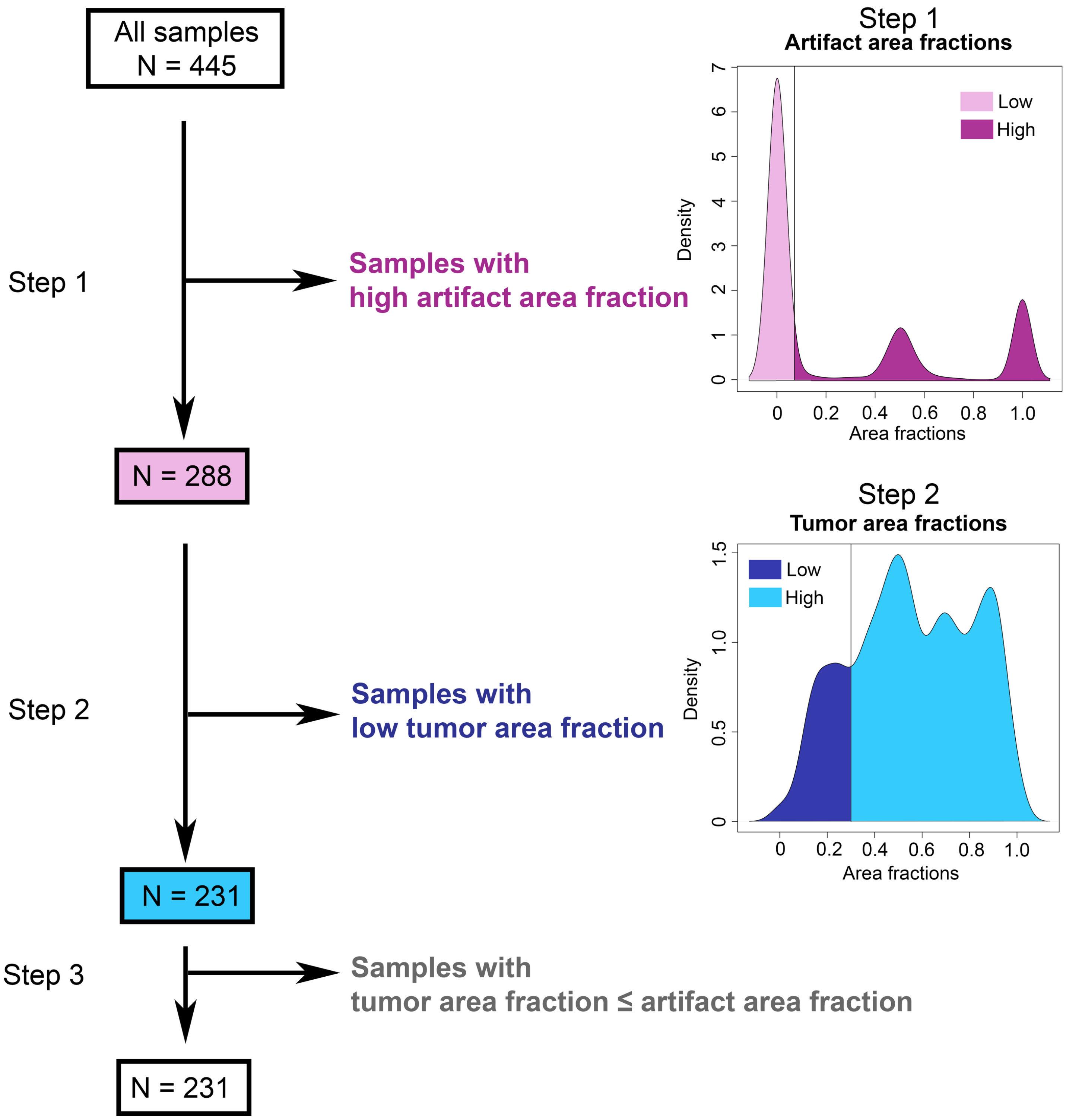

Figure S2. Characterization and filtering of the TMA

a.

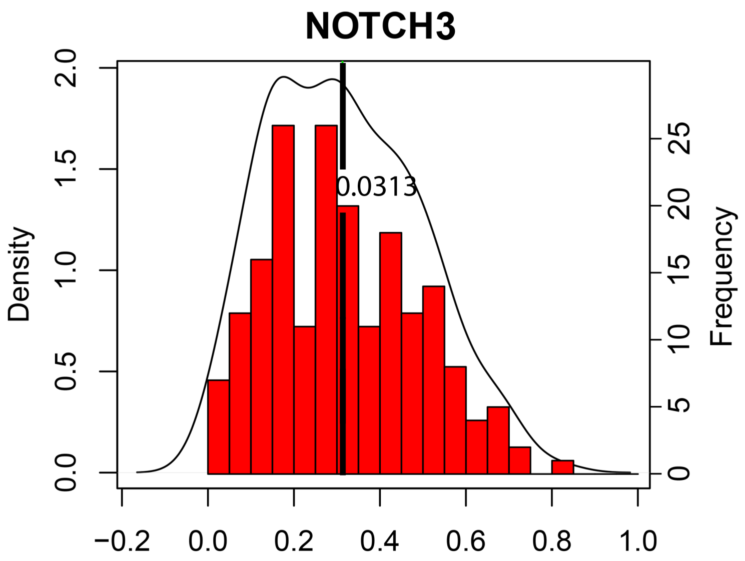

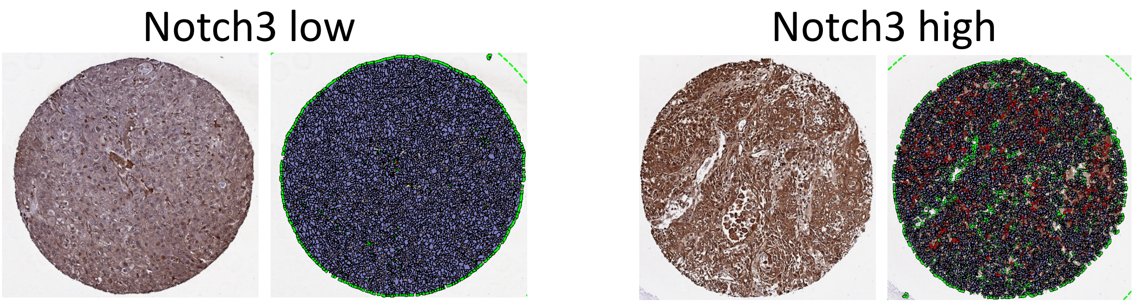

b.

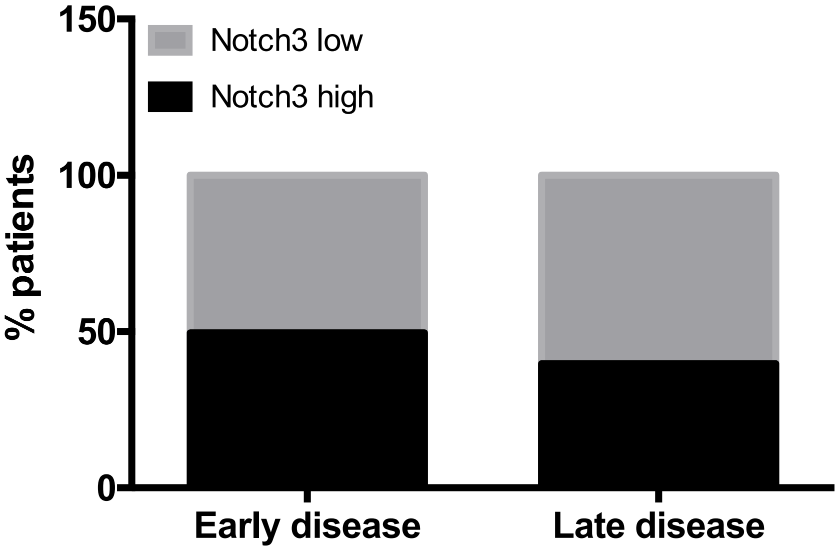

Figure S3. Notch3 expression in the TMA.

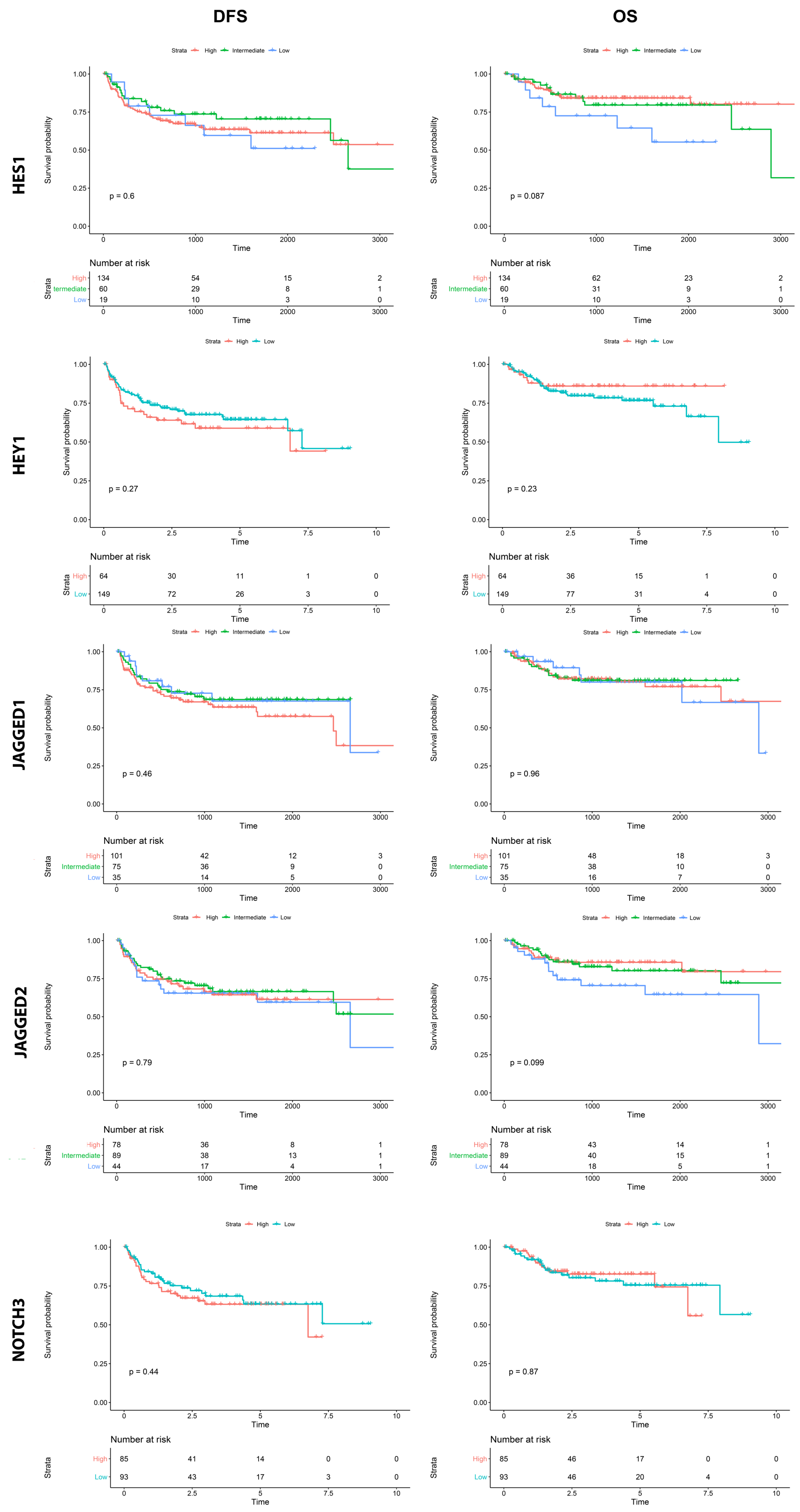

Figure S4. Overall and disease-free survival analysis based on expression of Notch pathway components

a.

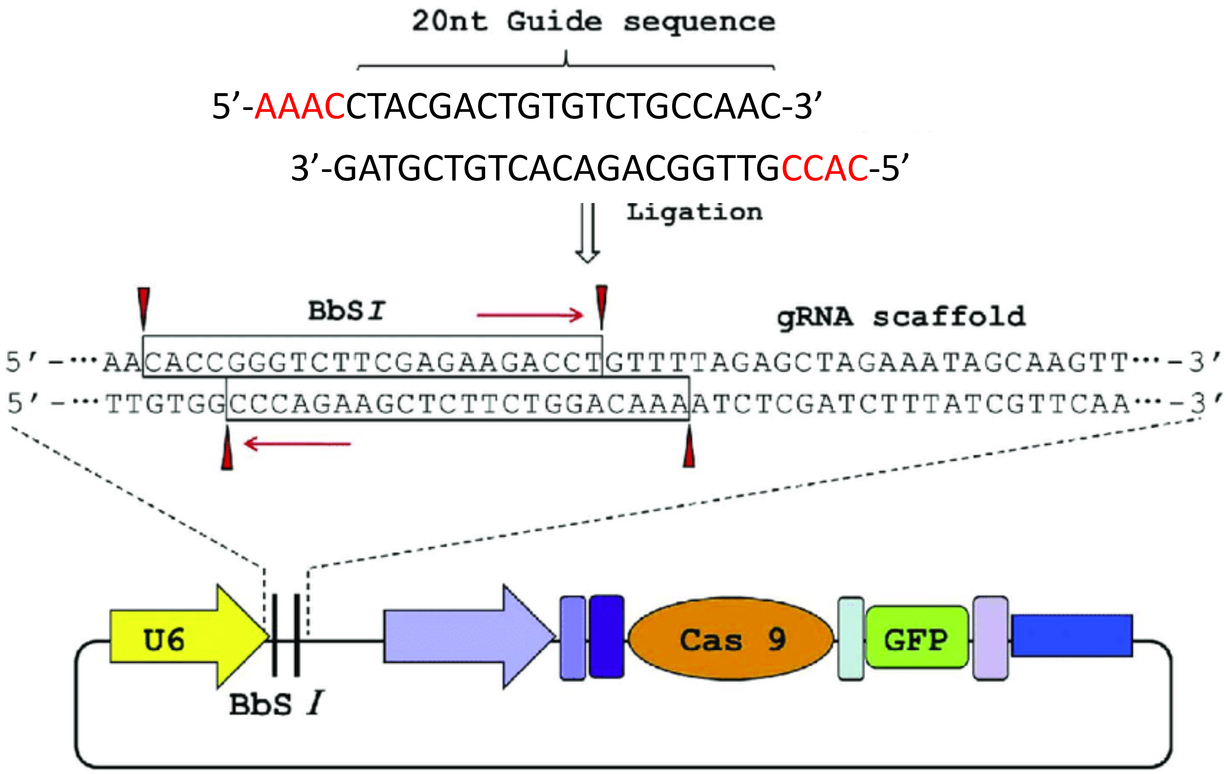

b.

5’-TCTGGGACCACAGGTGTGAACTGCGAAGTGAACATTGACGACTGTGCCAGCAACCCCTGCACCTTTGGAGTCTG
CCATGATGGCATCAACCGCTACGACTGTGTCTGCCAACCTGGCTTCACAGGGCCCCTTTGTAACGTGGAGATCAATG
AGTGTGCTTCCAGCCCATGCGGCGAGGGAGGTTCCTGTGTGGATG

c.

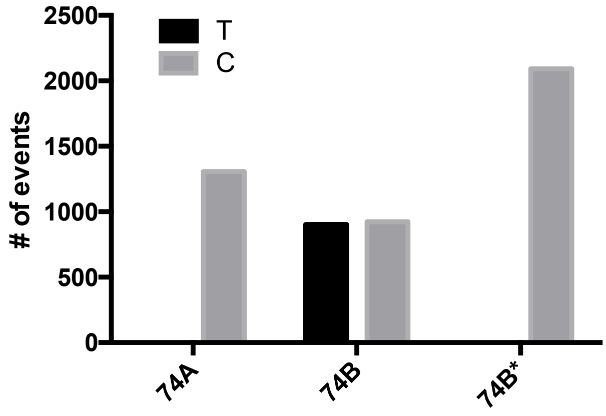

Figure S5. CRISPR/CAS9 system was utilized to correct Notch 3 mutation in UM-SCC-74B cells.

a.

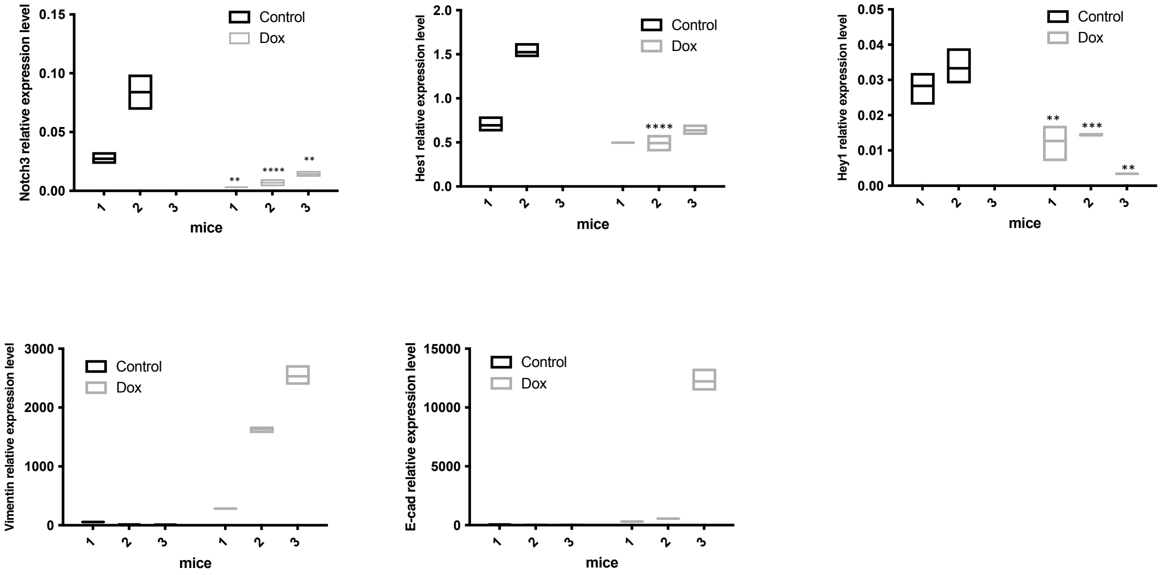

b.

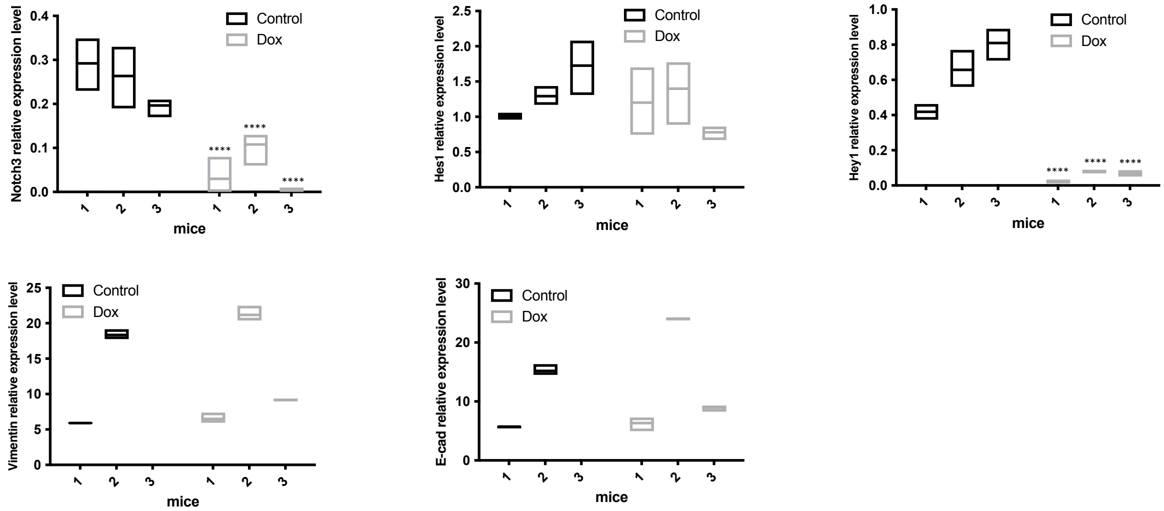

c.

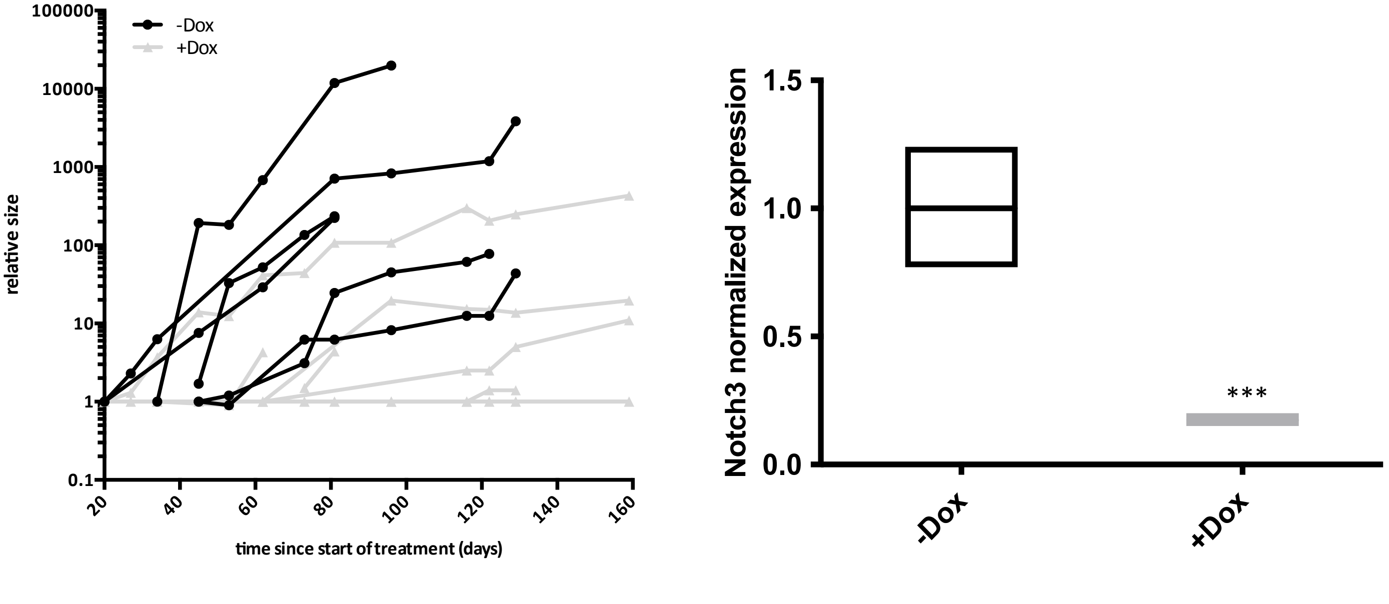

Figure S6. Growth quantification of lymph node metastasis and Notch knockdown confirmation in mice

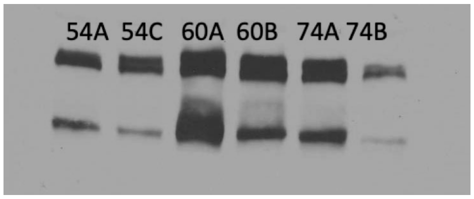
54A 54C 60A 60B 74A 74B

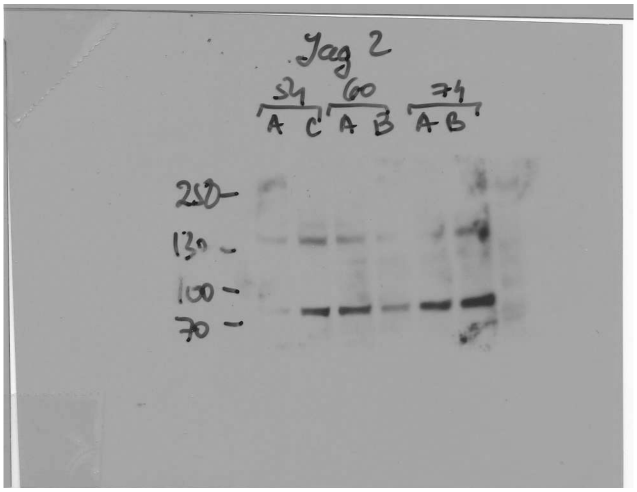
Jag 2
54
60
74
A C A B A B
250
130
100
70

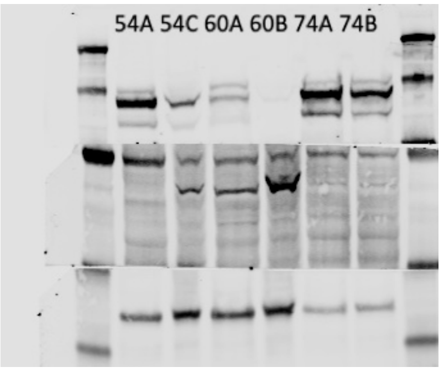

Figure S7. Uncropped blots for figure 2.

Supplementary table and figure legends

Figure S1 (related to Figures 2 and 3). High throughput fluorescent imaging was utilized to evaluate the effects of knocking down of Notch3 on the growth of HNSCC cells derived from primary tumors and metastases. a. Metastatic and primary lines from each set were labelled by infecting with lentivirus expressing either H2B-GFP or H2B-RFP, mixed in an equal ratio (300 cells/well of each line), plated in 384 well plates, and transfected with Dharmacon siRNA pools (using RNAmax Lipofectamine) against the target of interest. Fluorescent images were acquired using In Cell analyzer. b. Gene silencing in cells shown in B is confirmed by qPCR analysis. All qPCR data is normalized to HPRT expression and to the average expression levels of each gene in mock transfected cells (control) for each cell line. The data is presented as mean $\pm$ SEM.

Figure S2 (related to Figure 5). Characterization and filtering of the TMA. Workflow for filtering of the TMA samples. Step 1 – removing of samples with high artefact area. Step 1 insert (right): a distribution of artefact areas across the 445 samples. The filtering threshold is marked by a vertical black line; samples with a high artifact area are marked by a dark purple, samples with a lot of artefact area are marked by light purple. Step 2 – removing samples with low tumor area. Step 2 insert (right): a distribution of tumor areas across the 288 samples. The filtering threshold is marked by a vertical black line; samples with a high tumor area are marked by a light blue, samples with a low tumor area are marked by a dark blue. Only the samples that have higher tumor area compared to the artefact area remained for further analysis (step 3).

Figure S3 (related to Figure 5). Notch3 expression in the TMA. a. Distributions of fractions of positive cells for Notch3 across the TMA cores. Y-axis on the left axis indicates Density values, related to the density curve (the density curve is showing the smoothed distribution of the points along the x-axis). Y-axis on the right indicates frequency values related to the histogram. Thresholds between the subpopulations obtained by using EM algorithm (see Methods) are indicated with vertical black lines; the exact values of the thresholds are indicated near the corresponding lines. B. Pearson's Chi-squared tests were performed to examine the association between the pTstages (early and late) and the level of protein expression. No significant dependency was observed.

Figure S4 (related to Figure 5). Overall survival and disease-free survival analysis among different populations of patients based on the expression of Notch pathway components. Each plot shows Kaplan-Meier curves showing either overall or disease-free survival for patients exhibiting high, intermediate, and low levels of HES1, HEY1, JAGGED1 and JAGGED2. Patients who were alive without the disease at the time of the record were defined as disease free. Patients who passed away from unrelated causes were removed from the analysis.

Figure S5 (related to Figure 6). CRISPR/CAS9 system was utilized to correct Notch 3 mutation in UT-SCC-74B cells. a. Schematic representation for cloning of the sgRNAs into the pX458 plasmid containing Cas9 and the gRNA scaffold (pX458) (*85*). b. Sequence of the oligo used as a repair template to correct the mutation. Sequence targeted by sgRNA is highlighted in green and the corrected mutation (C) is labeled in red. c. Droplet digital PCR was performed using a custom dual-label Taqman assay designed to target the C allele (in VIC) and T allele (in FAM) at c.1939. In the graph, the Y axis denotes the number of droplets that are positive for 'T' or 'C'.

Figure S6 (related to Figures 7 and 8). Growth quantification of lymph node metastasis and Notch knockdown confirmation in mice. Expression of Notch pathway components and EMT

markers in subcutaneous tumors seeded by UT-SCC-74A (a) and UT-SCC-74B (b) cells from control and doxycycline treated mice was measured by qPCR. c. Growth of lymph node metastasis seeded by UT-SCC-74B cells was measured and quantified using the Xenogen IVIS-200 instrument. d. Levels of Notch3 RNA in UT-SCC-74B seeded lymph node metastasis from control and doxycycline treated mice were measured using qPCR. Average expression from 3 independent tumors is plotted.

Figure S7 (related to Figure 2). Uncropped blots.
