# Supplementary material for: Identification of acquired Notch3 dependency in metastatic Head and Neck Cancer
Source: Commun Biol. 2023 May 18;6:538. doi: 10.1038/s42003-023-04828-9 (PMC10195806; doi:10.1038/s42003-023-04828-9)
Supplement: Supplementary file 3 — Description of Additional Supplementary Files [file 42003_2023_4828_MOESM3_ESM.pdf]

Description of Additional Supplementary Files

File name: Supplementary Data 1
Description: The top ranked genes mutated in metastatic cells compared to primary tumors cells are presented (196 genes, q value < 10^-3). To obtain metastatic-specific somatic mutation calls, we performed mutation calling for metastatic cell lines, using primary samples as matched normal in Mutect run. We then used Oncotator (10.1002/humu.22771) to annotate the somatic mutations from Mutect. Finally, we performed additional filtering (using Oncotator's annotations for Exome Sequencing Project and dbSNP databases) to further filter germline mutations.

File name: Supplementary Data 2
Description: Cell lines derived from HNSCC patients used in this study are listed including relevant clinical characteristics.

File name: Supplementary Data 3
Description: List of genes used for targeted sequencing panel.

File name: Supplementary Data 4
Description: The data was considered with patient and tumor site as conditions and a one-way ANOVA with a Benjamini-Hochberg FDR corrected p<0.05 and post-hoc test (Tukey HSD test) for specific comparison differences was used. The significant probes were then filtered to find only those that were at least 1.5-fold differentially regulated in all 3 sets of lines primary vs. metastasis. A Benjamini and Yekutieli corrected (p<0.1) hypergeometric test, to look for enriched Gene Ontology categories that may overlap, was applied to each of these lists in turn.

File name: Supplementary Data 5
Description: siMEM hierarchical regression algorithm was applied to test for differences in gene essentiality between primary tumor and metastasis derived cells across the three sets of cell lines. The table shows dropout rate values in metastatic and primary tumor derived cells as well as differences in dropout rates between primary and metastatic cells for each gene.

File name: Supplementary Data 6
Description: Clinical information for HNSCC patients whose tumor samples were used to construct the TMA.

File name: Supplementary Data 7
Description: Examples from samples exhibiting various levels of Hes1, Hey1, Jagged1 (Jag1) and Jagged2 (Jag2) proteins. Levels are referred to as "low", "int" (intermediate) and "high". "Low for all" refers to a sample exhibiting low levels of all 4 proteins.

File name: Supplementary Data 8
Description: Crosstab analysis for demographic, clinical and histopathological data, including pathological grading and lymph node metastasis. The table contains age, gender (M = male; F = female), pTstage (stages 1,2,3 and 4), pNstage (stages 0,1,2,3 and Nx), pMstage (stage 0), Overall pathological stage (stages 1,2,3, and 4), survival status (1 = dead, 0 = alive), mets (1 = yes, 0 = no).

File name: Supplementary Data 9
Description: Survival analysis for overall and disease-Free survival. Each table presents results of univariate and multivariate analyses. Analyses were performed using Cox proportional hazards regression models.

File name: Supplementary Data 10
Description: qPCR primers, sgRNAs and repair Oligos used in this study are listed.

File name: Supplementary Data 11
Description: Source data for the graphs.

File name: Supplementary Data 12
Description: Source images for gels.
