# Supplementary material for: Identification of acquired Notch3 dependency in metastatic Head and Neck Cancer
Source: Commun Biol. 2023 May 18;6:538. doi: 10.1038/s42003-023-04828-9 (PMC10195806; doi:10.1038/s42003-023-04828-9)
Supplement: Supplementary file 15 — Reporting Summary [file 42003_2023_4828_MOESM15_ESM.pdf]

nature research

Corresponding author(s): Maria Kondratyev

Last updated by author(s): 10/17/2022

Reporting Summary

Nature Research wishes to improve the reproducibility of the work that we publish. This form provides structure for consistency and transparency in reporting. For further information on Nature Research policies, see our Editorial Policies and the Editorial Policy Checklist.

Statistics

For all statistical analyses, confirm that the following items are present in the figure legend, table legend, main text, or Methods section.

n/a	Confirmed	
☐	☒	The exact sample size (n) for each experimental group/condition, given as a discrete number and unit of measurement
☐	☒	A statement on whether measurements were taken from distinct samples or whether the same sample was measured repeatedly
☐	☒	The statistical test(s) used AND whether they are one- or two-sided<br>*Only common tests should be described solely by name; describe more complex techniques in the Methods section.*
☐	☒	A description of all covariates tested
☐	☒	A description of any assumptions or corrections, such as tests of normality and adjustment for multiple comparisons
☐	☒	A full description of the statistical parameters including central tendency (e.g. means) or other basic estimates (e.g. regression coefficient) AND variation (e.g. standard deviation) or associated estimates of uncertainty (e.g. confidence intervals)
☒	☐	For null hypothesis testing, the test statistic (e.g. F, t, r) with confidence intervals, effect sizes, degrees of freedom and P value noted<br>*Give P values as exact values whenever suitable.*
☒	☐	For Bayesian analysis, information on the choice of priors and Markov chain Monte Carlo settings
☒	☐	For hierarchical and complex designs, identification of the appropriate level for tests and full reporting of outcomes
☒	☐	Estimates of effect sizes (e.g. Cohen's d, Pearson's r), indicating how they were calculated

Our web collection on statistics for biologists contains articles on many of the points above.

Software and code

Policy information about availability of computer code

Data collection	GenomeStudio Version 2011.1 was used for the gene expression Ilumina microarrays.
Data analysis	BWA, muTect, Oncotator DAVID, MutSigCV, siMEM package, R environment 5.4.5, mclust package 3.6.0 , GenomeStudio Version 2011.1

For manuscripts utilizing custom algorithms or software that are central to the research but not yet described in published literature, software must be made available to editors and reviewers. We strongly encourage code deposition in a community repository (e.g. GitHub). See the Nature Research guidelines for submitting code & software for further information.

Data

Policy information about availability of data

All manuscripts must include a data availability statement. This statement should provide the following information, where applicable:
- Accession codes, unique identifiers, or web links for publicly available datasets
- A list of figures that have associated raw data
- A description of any restrictions on data availability

Raw data files for the Illumina Gene Expression analysis have been deposited in the NCBI Gene Expression Omnibus under the accession number GSE117753.

Field-specific reporting

Please select the one below that is the best fit for your research. If you are not sure, read the appropriate sections before making your selection.

☒ Life sciences ☐ Behavioural & social sciences ☐ Ecological, evolutionary & environmental sciences

For a reference copy of the document with all sections, see nature.com/documents/nr-reporting-summary-flat.pdf

Life sciences study design

All studies must disclose on these points even when the disclosure is negative.

Sample size	We used an accepted sizes based on what is accepted and published for similar types of experiments.
Data exclusions	For TMA analysis, cores that did not seem to contain tumor tissue were excluded from the analysis. The exact process of this exclusion can be provided upon acceptance.
Replication	All in vitro experiments were repeated at least 4 times. All reported results are reproducible.
Randomization	The mice were allocated to control and treatment groups through the process of randomization aiming to achieve a similar average of tumor size and the beginning of treatment
Blinding	During imaging of mice and image analysis of tumor histology, the analysts were blinded to groups of treatment and control.

Reporting for specific materials, systems and methods

We require information from authors about some types of materials, experimental systems and methods used in many studies. Here, indicate whether each material, system or method listed is relevant to your study. If you are not sure if a list item applies to your research, read the appropriate section before selecting a response.

Materials & experimental systems

n/a	Involved in the study	
☐	☒	Antibodies
☐	☒	Eukaryotic cell lines
☒	☐	Palaeontology and archaeology
☐	☒	Animals and other organisms
☒	☐	Human research participants
☒	☐	Clinical data
☒	☐	Dual use research of concern

Methods

n/a	Involved in the study	
☒	☐	ChIP-seq
☒	☐	Flow cytometry
☒	☐	MRI-based neuroimaging

Antibodies

Antibodies used

Jag1 Abcam Cat# ab7771
RRID: AB_2280547
Jag2 Abcam Cat# ab109627 RRID: AB_10860796
Actin Abcam Cat# ab8227 RRID: AB_2305186
Hey1 Novus Cat# NBP2-47436

Hes1 Novus Cat# NBP2-67642
HRP anti-mouse GE Healthcare Cat# NA931, RRID: AB_772210
HRP anti-rabbit GE Healthcare Cat# GENA934, RRID: AB_2722659

Validation

All the antibodies were used according to company recommendation.
Positive and negative controls were included where possible.
More information can be provided upon acceptance.

Eukaryotic cell lines

Policy information about cell lines

Cell line source(s)

UT-SCC-54A Gift of Dr. Grenman RRID: CVCL_7863
UT-SCC-54C Gift of Dr. Grenman NA
UT-SCC-60A Gift of Dr. Grenman RRID: CVCL_A089

	UT-SCC-60B Gift of Dr. Grenman RRID: CVCL_A090<br>UT-SCC-74A Gift of Dr. Grenman NA<br>UT-SCC-74B Gift of Dr. Grenman NA
Authentication	HNSCC patient-derived lines were generously provided by Dr. Grenman's lab, authenticated by STR and tested for mycoplasma and bacterial contamination.
Mycoplasma contamination	All cell lines tested negative for mycoplasma contamination
Commonly misidentified lines<br>(See ICLAC register)	n/a

Animals and other organisms

Policy information about studies involving animals; ARRIVE guidelines recommended for reporting animal research

Laboratory animals	NOD-PrkdcscidIl2rgem1Smoc Mus musculus (IMSR Cat# NM-NSG-001, RRID: IMSR_NM-NSG-001) were bred in house and used for generation of tongue tumors as described.
Wild animals	n/a
Field-collected samples	n/a
Ethics oversight	All animal experimentation was conducted in accordance with the Canadian Guide for the Care and Use of Laboratory Animals, and protocols were approved by the Animal Care Committee at the Princess Margaret Hospital Cancer Center, University Health Network, University of Toronto (Toronto, Ontario, Canada)

Note that full information on the approval of the study protocol must also be provided in the manuscript.
